# Visible Light-Mediated Sustainable Antibacterial Activity and Osteogenic Functionality of Au and Pt Multi-Coated TiO_2_ Nanotubes

**DOI:** 10.3390/ma14205976

**Published:** 2021-10-11

**Authors:** Kyoung-Suk Moon, Young-Bum Park, Ji-Myung Bae, Eun-Joo Choi, Seung-Han Oh

**Affiliations:** 1 Department of Dental Biomaterials, The Institute of Biomaterial and Implant, School of Dentistry, Wonkwang University, Iksan 54538, Korea; ksemoon@hanmail.net (K.-S.M.); baejimy@wku.ac.kr (J.-M.B.); 2 Department of Prosthodontics, School of Dentistry, Yonsei University, Seoul 03722, Korea; drybpark@yuhs.ac; 3 Department of Oral and Maxillofacial Surgery, School of Dentistry, Wonkwang University, Iksan 54538, Korea

**Keywords:** gold, platinum, multi-coating, titania nanotubes, visible light, antimicrobial activity, osteogenic functionality

## Abstract

The visible light reactions of noble metal-based photocatalysts have been increasingly utilized to investigate their antibacterial activities. Furthermore, the photoreactions at various visible light wavelengths for specific combinations of titania nanotubes and noble metal nanoparticles have been found to promote osteogenic functionality. In this investigation, a novel multi-coating combination of noble metals (gold and platinum) on titania nanotubes was assessed using plasmonic photocatalysis and low-level laser therapy at 470 and 600 nm. The results showed that this coating on the nanotubes promoted antibacterial activity and osteogenic functionality. The order in which the gold and platinum coatings were layered onto the titania nanotubes strongly affected the osteogenic performance of the human mesenchymal stem cells. These results have identified a new approach for the development of efficient novel combinations of noble metal nanoparticles and titania nanotubes with visible light responses, sustainable antimicrobial activity, and osteogenic functionality.

## 1. Introduction

Ideal implant success in dentistry occurs when there is stable functionality of the dental prosthesis, which results from a strong bonding force based on high-quality osseointegration without infection in the region of the peri-implant soft tissue. If there is total implant failure, the desired function and aesthetics are not achieved, for mechanical or biological reasons [1,2]. Implant failure can be classified as early, which occurs during the initial loading period, or delayed, which occurs after the osseointegration process has been completed and implant function is achieved. Bacterial infection is one of the factors that cause early failure and may occur due to contamination of the implant itself, infection during surgery, or improper healing [3].

To improve the limitations of conventional antibacterial treatment methods such as a direct injection of antibacterial drugs, new technologies such as antimicrobial biomolecules [4,5,6], antibacterial nanoparticles [7,8,9], photodynamic therapy [10,11,12], and photocatalytic therapy [13,14,15] have been introduced. Among these, titania (TiO_2_) based photocatalytic antibacterial treatments are predicted to be useful for dental and medical implants based on titania or titania alloys.

However, titania has the limitation of expressing a photocatalytic effect in the ultraviolet region due to its high bandgap energy. Many studies have attempted to address this limitation by expanding the photocatalytic effects to the visible light region [16,17,18]. Localized surface plasmon resonance (LSPR) related to the plasmonic photocatalyst generated by the combination of noble metals and titania is expected to be safe for use in implant surface treatment due to its excellent levels of biocompatibility [19,20,21]. 

In previous studies, we have developed Au and Pt single-coated TiO_2_ nanotubes showing visible light photocatalytic activity and osteogenic acceleration with selective visible light irradiation [22,23,24]. This research also showed that the resulting species of noble metal nanoparticles affected the antibacterial activity and osteogenic performance and were the basis for further investigations into variable factors such as species, morphology, and the coating conditions of the noble metal nanoparticles. Based on these factors, the multi-coating of noble metal nanoparticles is expected to double its properties and strongly express the bandgap features of each metal. Furthermore, previous studies related to Au and Pt bimetallic nanoparticles reported that Pt nanoparticles prevent the coarsening of Au nanoparticles, thereby exhibiting an improved and stable plasmonic photocatalytic effect [25,26].

In this study, we prepared Au and Pt multi-coated TiO_2_ nanotubes and estimated the effect of the stacking sequence of Au and Pt on the antibacterial activity based on plasmonic photocatalysis and the osteogenic capability of the Au and Pt multi-coated TiO_2_ nanotubes under in vitro conditions. We also evaluated the osteogenic performance of Pt-coated TiO_2_ nanotubes showing excellent osteogenic function in a previous study and Au and Pt multi-coated TiO_2_ nanotubes.

## 2. Materials and Methods

### 2.1. Preparation of Au and Pt Multi-Coated TiO_2_ Nanotubes

The procedure, materials, and reagents for preparing anatase crystalline phased TiO_2_ nanotubes with diameters of 100 nm are the same as our previous research [24]. The specimen for the anodization process was 5 × 5 cm^2^ and was cut to the size of 1 × 1 cm^2^ to perform noble metal coating and in vitro experiments. Au and Pt multi-coatings were applied to the TiO_2_ nanotubes using an ion plasma sputter (E-1030; Hitachi Co., Tokyo, Japan). 

For this investigation, we designated AuPt-TiO_2_ nanotubes (condition for the first coating of Au and the second coating of Pt) and PtAu-TiO_2_ nanotubes (condition for the first coating of Pt and the second coating of Au) according to the coating sequence of the Au and Pt on the TiO_2_ nanotubes. The coating time of Au and Pt were 30 and 60 s, respectively. TiO_2_ nanotubes without noble metal coating were used as a control.

### 2.2. Surface Characteristics

The equipment and measurement methods such as field-emission scanning electron microscope (FE-SEM; S4800S; Hitachi & Horiba Co., Tokyo, Japan), transmission electron microscope (TEM; JEM-ARM200F; JEOL Co., Tokyo, Japan), and diffuse reflectance UV-Vis-NIR spectrophotometer (SolidSpec-3700; Shimadzu Co., Tokyo, Japan) used in this study for the surface characterization of the Au and Pt multi-coated TiO_2_ nanotubes are the same as in our previous study [24]. The X-ray photoelectron spectroscopy (XPS; K-Alpha; Thermo Fisher Scientific Inc., Waltham, MA, USA) was equipped with a monochromatic A1 Kα source. From the FE-SEM images, the diameter of forty nanoparticles coated on the TiO_2_ nanotubes was measured using Image J software (Version 1.53b, NIH, Bethesda, MD, USA) to evaluate the size distribution of deposited nanoparticles. 

### 2.3. Antibacterial Activity Test

The experimental conditions and procedures of the antibacterial activity test are the same as those of our previous study except for the period of visible light irradiation [24]. We used Staphylococcus aureus (*S. aureus*; ATCC 25923, Manassas, VA, USA) to perform the antibacterial activity test. All specimens in this experiment were autoclaved at 120 °C for 10 min. We performed visible light irradiation on the *S. aureus* for 15 min using a lab-fabricated 470 and 600 nm visible light-emitting diode (LED) device (Power density = 5.5 mW/cm^2^, the distance between the specimen and the LED device = 2 cm). After light irradiation, we collected 100 μL bacteria from the specimen by sonication for 5 min (SH-2100; Saehan Ultrasonic Co., Seoul, Korea). The dilution concentration of the collected bacteria was adjusted by adding PBS solution. One hundred microliters of the diluted bacteria were seeded on an agar layer in a 100 mm cell culture dish. After 24 h of additional incubation, the value of the antibacterial activity is expressed by multiplying the number of attached bacteria by the dilution index. Compared with a previous study [24], the irradiation time was reduced from 30 to 15 min to prevent unwanted bacteria death due to drying the agar culturing the bacteria during a long irradiation time. To minimize the side effect of unwanted light irradiation, all samples were placed in the dark during the entire experimental period.

### 2.4. Human Mesenchymal Stem Cell Cultures

The cell culture conditions, growth media, and osteogenic differentiation media of human mesenchymal stem cells (hMSCs; Lonza Co., Basel, Switzerland) used in this study are the same as in our previous study [24]. The cultured hMSCs were washed by phosphate buffer solution (PBS; Invitrogen Co., Waltham, MA, USA) and replaced with fresh media every 3 days.

### 2.5. Alkaline Phosphatase (ALP) Activity Assay

The ALP activity assay was performed by ALP colorimetric assay kit (K412; Biovision Inc., Milpitas, CA, USA) composed of ALP assay buffer, 5 mM para-nitrophenyl phosphate (p-NPP), ALP enzyme, and stop solution. We used a 24-well plate to place the specimen and seeded 20,000 cells of hMSCs onto each well. After 1 h of incubation, we performed 1st visible light irradiation on the hMSCs for 30 min using lab-fabricated visible light LED device. Further visible light irradiations were conducted after changing the osteogenic media every 3 days. After 1 and 2 weeks of cell culture, the hMSCs were collected and lysed in a 200 μL of Assay buffer solution. After 30 min of lysis, 50 μL of 5 mM p-NPP solution was added to 80 μL cell lysate and reacted at 37 °C for 30 min. Then, 20 μL of stop solution was added to the reaction solution to stop the p-NPP reaction. The absorbance value of the final solution was measured by the microplate ELISA reader (SpectroMax 250; Thermo Electron Co., Waltham, MA, USA) at 405 nm wavelength. The value of ALP activity was calculated by dividing the absorbance value of each sample by the total protein amount of each sample. The quantification of proteins was conducted by Pierce^TM^ BCA Protein Assay Kit (Thermo Fisher Scientific Inc., Waltham, MA, USA). To minimize the experimental errors, three specimens were combined at once to make one sample. The number of samples for statistical analysis was four.

### 2.6. hMSC Morphological Changes under Visible Light Irradiation

The filopodia behavior of the hMSCs cultured on the experimental specimen after visible light irradiation was observed by FE-SEM. We used a 24-well plate to place the specimen and seeded 5000 cells of hMSCs onto each well. After 24 h of cell culture, we irradiated 470 and 600 nm visible light onto the specimen for 30 min. The procedure and reagents for fixing, dehydrating, and drying the hMSCs cultured on the specimen are the same as in our previous study [24]. The micromorphological changes of the hMSCs were checked by FE-SEM.

### 2.7. Quantitative Real-Time PCR Assay

The real-time PCR assay equipment, experimental procedures, reagents, and the TaqMan® PCR primers (GAPDH, OPN, and BSP) used in this study are the same as in the previous study [24]. In addition, the TaqMan® PCR primer for OCN (Hs01587813_g1, Amplicon length: 166) was also used to analyze the osteocalcin gene expression. The culture procedure of the hMSCs and visible light irradiation conditions for the quantitative real-time PCR assay were the same as those for the ALP activity assay. In brief, after 1 and 2 weeks of cell culture, the total RNA was extracted from the hMSCs and reverse-transcribed into cDNA. The expressions of interest genes and GAPDH (house-keeping gene) were obtained from real-time PCR assay using 1 μL cDNA. To minimize the experimental errors, three specimens were combined at once to make one sample. The number of samples for statistical analysis was six. 

### 2.8. Alizarin Red Assay

The culture procedure for the hMSCs and visible light irradiation conditions for the alizarin red assay were the same as those for the ALP activity assay. After 2 and 3 weeks of cell culture, the specimen was rinsed with PBS solution and fixed with 4% paraformaldehyde solution (Sigma-Aldrich Co., St. Louis, MO, USA) at 25 °C for 20 min. The fixed specimen was then stained with alizarin red solution (Sigma) for 20 min. The stained specimen was washed with de-ionized water and reacted with 10% acetic acid (JT Baker Co., Phillipsburg, NJ, USA) for 30 min. The reacted specimen was stored at 85 °C for 10 min. The supernatant was then collected from the specimen after the centrifugation (11,000 rpm for 15 min: Micro 17TR, Hanil Co., Daejeon, Korea). Neutralization was performed by adding the same volume of 10% ammonium hydroxide (Sigma-Aldrich Co., St. Louis, MO, USA) to the supernatant. The absorbance value of the neutralized supernatant was measured at 405 nm with a microplate ELISA reader. To minimize the experimental errors, three specimens were combined at once to make one sample. The number of samples used in the statistical analysis is three. 

### 2.9. Statistical Analysis 

All data obtained by conducting this study were expressed as means standard deviations. Data analysis was performed by one-way analysis of variance (one-way ANOVA; IBM SPSS Statistics 24.0; IBM, Armonk, NY, USA) and Duncan’s multiple range test as a post hoc. A *p*-value less than 0.05 is statistically significant.

## 3. Results

### 3.1. Surface Characteristics

#### 3.1.1. FE-SEM Observation

**Figure 1:**
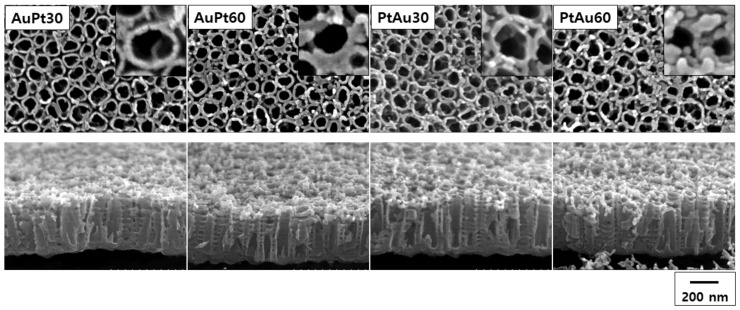
FE-SEM characterization of AuPt-TiO_2_ nanotubes and PtAu-TiO_2_ nanotubes (AuPt30 and AuPt60; 30 and 60 s of Au and Pt sputtering in this order, PtAu30 and PtAu60; 30 and 60 s of Pt and Au sputtering in this order, respectively) (top: plain view, bottom: oblique view, inset: magnified plain view).

The FE-SEM images for the AuPt-TiO_2_ nanotubes and PtAu-TiO_2_ nanotubes after 30 and 60 s of plasma coating are shown in Figure 1. There were no significant changes in the morphology of the surface layer regardless of the stacking order of the Au and Pt. However, the large agglomeration of the nanoparticles was observed in the experimental group coated for 60 s. The average diameter of the AuPt-TiO_2_ nanotubes and PtAu-TiO_2_ nanotubes for 30 s coating was 9.89 ± 1.68 and 12.76 ± 2.91 nm, and when coated for 60 s it was 21.32 ± 4.27 and 19.93 ± 3.80 nm, respectively.

#### 3.1.2. Diffuse Reflectance UV-Vis-NIR Spectrophotometry

**Figure 2:**
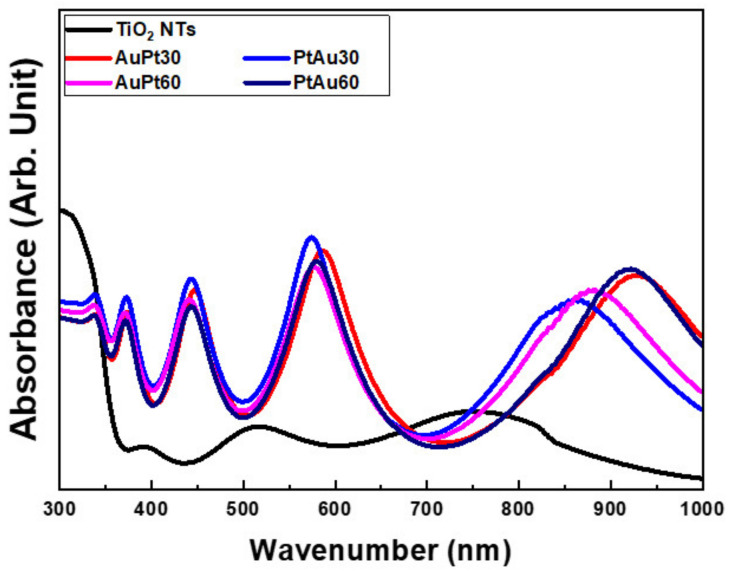
UV-Vis-NIR diffuse reflectance spectrophotometric curves for AuPt–TiO_2_ nanotubes and PtAu–TiO_2_ nanotubes after various sputtering periods (AuPt30 and AuPt60; 30 and 60 s of Au and Pt sputtering in this order, PtAu30 and PtAu60; 30 and 60 s of Pt and Au sputtering in this order, respectively).

UV-Vis-NIR diffuse reflectance spectrophotometric curves for the AuPt-TiO_2_ nanotubes and PtAu-TiO_2_ nanotubes are shown in Figure 2. The four major absorption spectra of the AuPt-TiO_2_ nanotubes and PtAu-TiO_2_ nanotubes were all found within the following wavelengths: 360–380, 420–480, 550–650, and 800–950 nm. Two peaks at 420–480 and 550–650 nm follow the LSPR based plasmonic photocatalysis and the photothermal scattering of Au and Pt multi-coated on TiO_2_ nanotubes, respectively [27]. Additionally, from the UV-Vis-NIR diffusion reflectance spectrophotometric results, there was no difference according to the stacking sequence of the Au and Pt coating. From the FE-SEM observations and diffuse reflectance UV-Vis-NIR spectra results, the Au and Pt coating time of 30 s was selected for other experiments to prevent the coarsening and agglomeration of the deposited nanoparticles and to optimize the experimental process for the visible light-mediated sustainable antibacterial activity and osteogenic differentiation. 

#### 3.1.3. TEM Observation and Elementary Analysis

**Figure 3:**
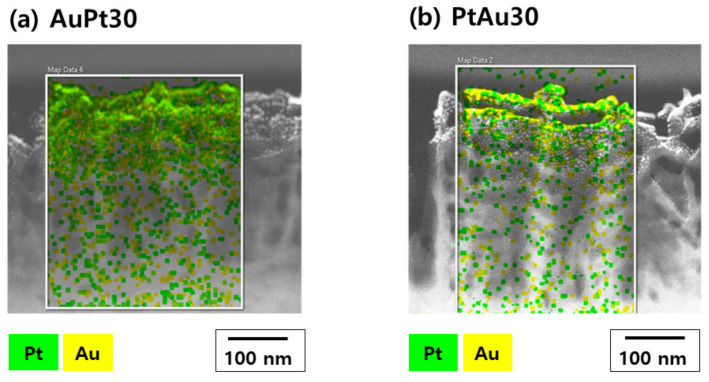
TEM characterization of (**a**) AuPt–TiO_2_ nanotubes (AuPt30) and (**b**) PtAu–TiO_2_ nanotubes (PtAu30).

TEM images of AuPt-TiO_2_ nanotubes and PtAu-TiO_2_ nanotubes are shown in Figure 3. From the elementary mapping of the TEM images, most of the Au and Pt nanoparticles were distributed on the upper layer of the TiO_2_ nanotubes. Additionally, 2.20 and 1.69 wt% of the Pt and 4.65 and 5.39 wt% of the Au were detected in the mapped area of the AuPt-TiO_2_ nanotubes and PtAu-TiO_2_ nanotubes, respectively. 

#### 3.1.4. XPS Analysis

**Figure 4:**
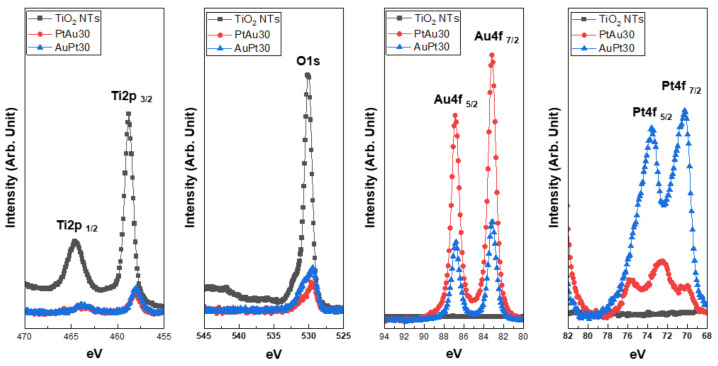
Ti 2p, O 1s, Au 4f, and Pt 4f XPS spectra of Au and Pt multi-coated TiO_2_ nanotubes.

The XPS spectra for the AuPt-TiO_2_ nanotubes and PtAu-TiO_2_ nanotubes are illustrated in Figure 4. The lower shift in the binding energy of the major peaks of Ti 2p and O 1s was caused by the decrease in Ti and O species following the coating of Au and Pt nanoparticles at the top surface of the TiO_2_ nanotubes. Moreover, a new peak at 72.5 eV was detected in the Pt 4f spectra of the PtAu30. This peak seemed to be the formation of Pt-O bonds when a small amount of Pt was first coated at the surface of the TiO_2_ nanotubes [28,29].

### 3.2. Antibacterial Activity Test

**Figure 5:**
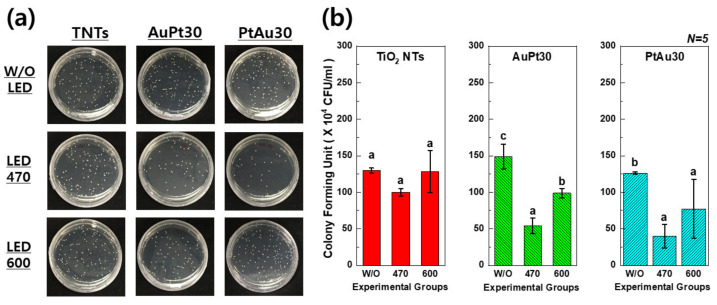
(**a**) Photo images of agar-based antibacterial activity test and (**b**) CFU values per unit volume of *S. aureus*. In the graph, W/O, 470, and 600 designate no irradiation, 470 nm irradiation, and 600 nm irradiation, respectively. Moreover, in each graph, the values of experimental groups with the same lowercase letter (a, b, or c) are not statistically different as determined by one-way ANOVA at α = 0.05.

In Figure 5, the CFU values of the Au and Pt multi-coated TiO_2_ nanotubes with 470 and 600 nm of visible light irradiation were significantly lower than those without visible light irradiation (*p* < 0.05). This result means that the combination of Au and Pt multi-coated TiO_2_ nanotubes and visible light irradiation showed antibacterial activity. 

### 3.3. Biocompatibility and Osteogenic Functionality of hMSCs

#### 3.3.1. hMSCs Morphological Observation by FE-SEM

**Figure 6:**
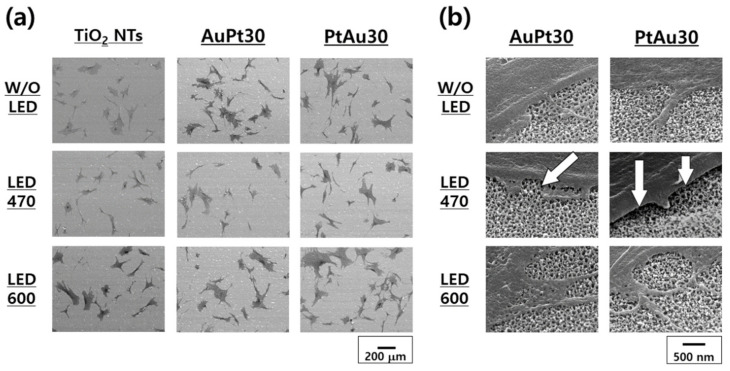
The morphological characterization of hMSCs cultured on Au and Pt multi-coated TiO_2_ nanotubes with and without visible light irradiation (470 and 600 nm for 30 min) was observed by FE-SEM ((**a**) 100× and (**b**) 50,000×).

The FE-SEM images (100× and 50,000×) of the hMSCs cultured on Au and Pt multi-coated TiO_2_ nanotubes with or without visible light irradiation (470 and 600 nm for 30 min) are shown in Figure 6. Many filopodia and spreading of the hMSCs were found in the experimental group irradiated with 600 nm light (Figure 6a). In Figure 6b, the filopodia of the hMSCs were detached from the surface with 470 nm light irradiation. However, the hMSCs irradiated with 600 nm light adhered well to the specimen surface and showed elongated filopodia.

#### 3.3.2. ALP Activity Assay

**Figure 7:**
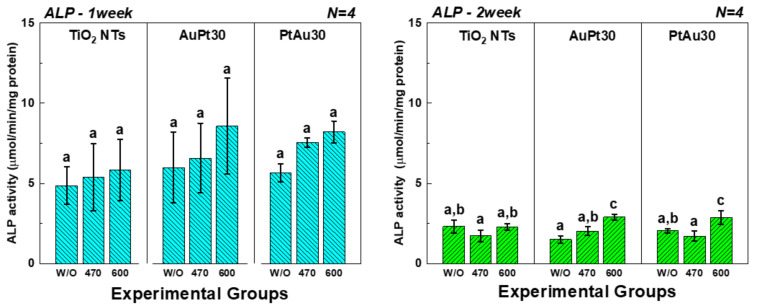
The ALP activity of the hMSCs cultured on Au and Pt multi-coated TiO_2_ nanotubes. In the graph, W/O, 470, and 600 designate no irradiation, 470 nm irradiation, and 600 nm irradiation, respectively. Moreover, in each graph, the values of experimental groups with the same lowercase letter (a, b, or c) are not statistically different as determined by one-way ANOVA at α = 0.05.

In Figure 7, after 1 week of cell culture, the ALP activity of AuPt-TiO_2_ nanotubes and PtAu-TiO_2_ nanotubes with 600 nm visible light irradiation was higher than that of the other experimental groups. After 2 weeks of cell culture, the ALP activity was decreased compared to the ALP activity of the first week. In addition, the 600 nm visible light irradiation still promoted the ALP activity of the experimental groups of AuPt-TiO_2_ nanotubes and PtAu-TiO_2_ nanotubes.

#### 3.3.3. Quantitative Real-Time PCR Assay

**Figure 8:**
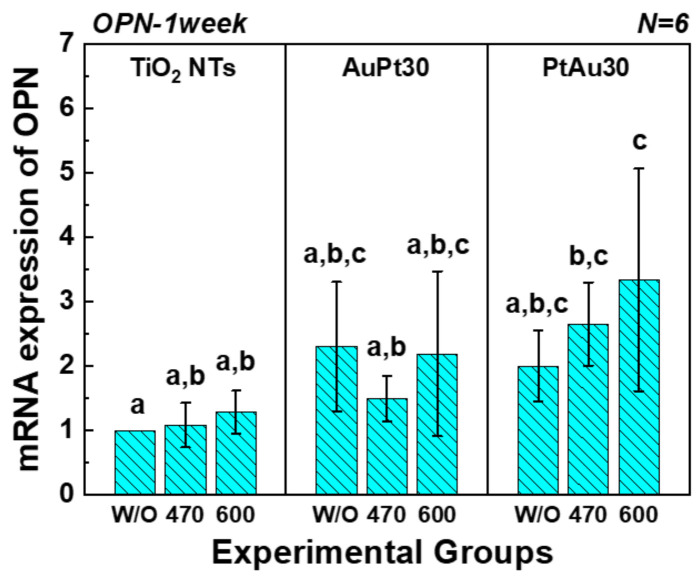

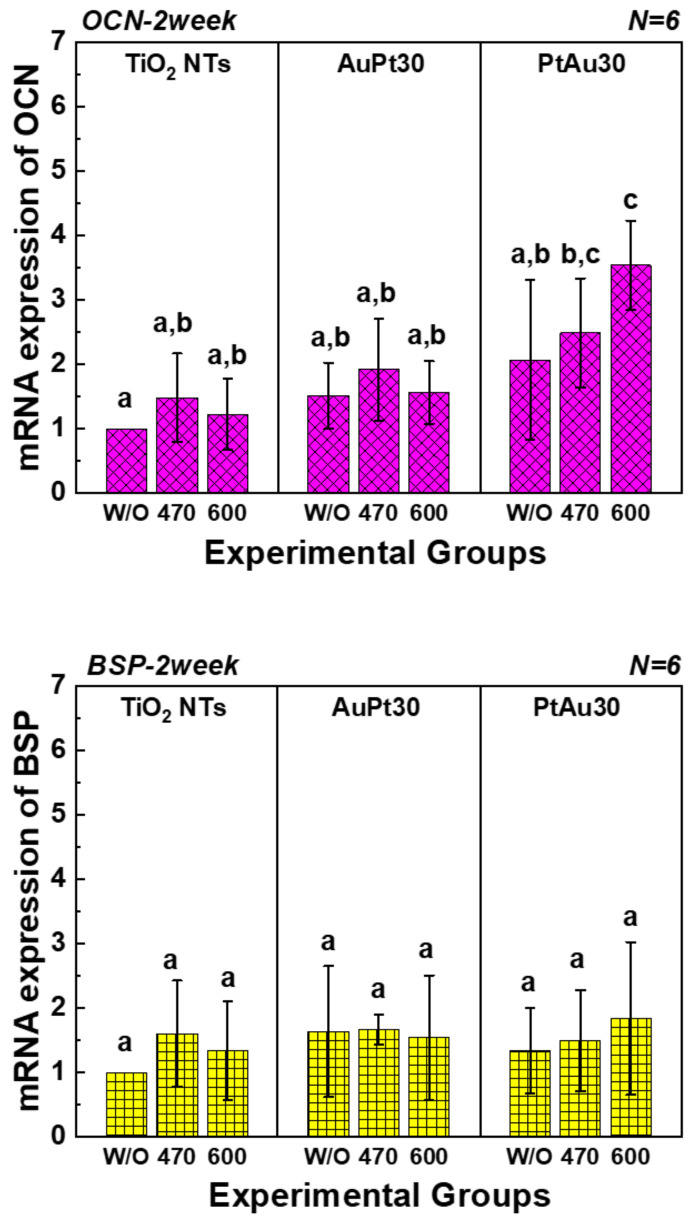
The mRNA expression of OPN, OCN, and BSP of the hMSCs cultured on Au and Pt multi-coated TiO_2_ nanotubes analyzed by quantitative real-time PCR assay. In the graph, W/O, 470, and 600 designate no irradiation, 470 nm irradiation, and 600 nm irradiation, respectively. Moreover, in each graph, the values of experimental groups with the same lowercase letter (a, b, or c) are not statistically different as determined by one-way ANOVA at α = 0.05.

The mRNA gene expression of OPN, OCN, and BSP of the hMSCs cultured on Au and Pt multi-coated TiO_2_ nanotubes analyzed by quantitative real-time PCR assay is shown in Figure 8. There was no statistical difference in mRNA expression of BSP between the groups. In addition, the mRNA expression of OPN and OCN of PtAu-TiO_2_ nanotubes with 600 nm visible light irradiation showed the highest values among all experimental groups and conditions. 

#### 3.3.4. Alizarin Red Assay

**Figure 9:**
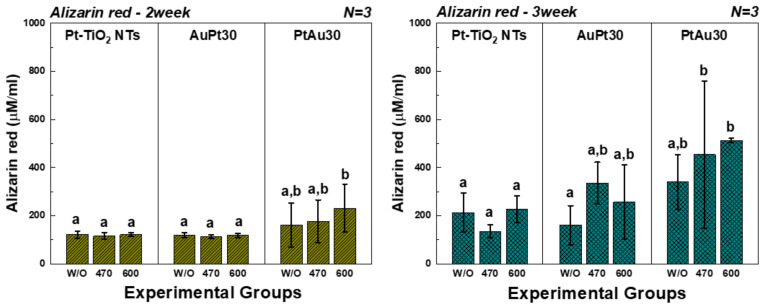
The alizarin red results of hMSCs cultured on Au and Pt multi-coated TiO_2_ nanotubes. In the graph, W/O, 470, and 600 designate no irradiation, 470 nm irradiation, and 600 nm irradiation, respectively. Moreover, in each graph, the values of experimental groups with the same lowercase letter (a or b) are not statistically different as determined by one-way ANOVA at α = 0.05.

The results of the alizarin red assay after 2 and 3 weeks of cell culture are shown in Figure 9. After 2 weeks of cell culture, the alizarin red values of the PtAu-TiO_2_ nanotubes were significantly higher than those of Pt-TiO_2_ nanotubes with 600 nm visible light irradiation (*p* < 0.05). In addition, after 3 weeks of cell culture, the alizarin red values of the PtAu-TiO_2_ nanotubes were significantly higher than those of Pt-TiO_2_ nanotubes on both conditions of the 470 and 600 nm visible light irradiation (*p* < 0.05).

## 4. Discussion

The FE-SEM images confirmed that the agglomeration and coarsening of the sputtered nanoparticles were formed at the surface of the TiO_2_ nanotubes coated with Au and Pt for 60 s, respectively. This phenomenon’s occurrence with the Au nanoparticles was relatively more frequent than with the Pt nanoparticles due to the differences in the rates of their ion plasma coatings [30]. In addition, from the inset images of Figure 1, the morphology of the nanoparticles changed from round to a rod or crescent shape during the ion plasma coating process. 

The diffuse UV-Vis-NIR spectrophotometry confirmed that four major absorbance peaks were detected at 360–380 nm (typical transition of electrons from the valence band to conduction band of anatase TiO_2_ after UV light irradiation [31]), 420–480 nm (plasmonic photocatalysis of Au and Pt coated TiO_2_ [32,33]), 550–650 nm (the short axis photothermal scattering of coated Au and Pt nanoparticles), and 800–950 nm (the long axis photothermal scattering of coated d Au and Pt nanoparticles). The wavelength range of four absorbance peaks is related to photothermal scattering and depends on the morphology of the Au and Pt nanoparticles deposited on the TiO_2_ nanotubes, as observed by FE-SEM images [34,35,36,37]. Moreover, there was no change in the range of the four major absorption wavelengths with the different Au and Pt coating periods. 

The TEM and elementary mapping images confirmed that most of the second-coated nanoparticles were distributed on the top layer of the nanotubes. In addition, the amount of coated Au was 2–3 times higher than the amount of coated Pt. These results showed a similar trend as those from the FE-SEM observations and EDX analysis. 

The XPS analysis confirmed that Ti 2p and O 1s peaks of Au and Pt multi-coated TiO_2_ nanotubes moved to lower energies compared to those of uncoated TiO_2_ nanotubes. This result means that the amounts of Ti and O species were decreased at the surface of the TiO_2_ nanotubes after Au and Pt coating [38,39]. In addition, the Pt 4f XPS spectra of PtAu-TiO_2_ nanotubes showed three peaks compared to those of Au-Pt TiO_2_ nanotubes having two peaks. This phenomenon is related to the amount of Pt deposited directly on the TiO_2_ nanotubes. Because Pt sputtering time was short (30 s), the small amounts of Pt nanoparticles were coated on the surface of the TiO_2_ nanotubes (Figure 1), and these nanoparticles are likely to be easily oxidized to form PtO_X_ structures [28,29]. Thus, the new binding peak (~72.5 eV) was expected to be the Pt-O bond generated by binding Pt metals to the TiO_2_ nanotubes in a short time. 

The antibacterial activity test confirmed that the antibacterial effects were generated by irradiation with visible light at 470 and 600 nm in both the AuPt-TiO_2_ nanotubes and PtAu-TiO_2_ nanotubes experimental groups. This phenomenon is presumed to be due to the expansion of the plasmonic photocatalyst and the shape change in the Au and Pt nanoparticles, showing a similar tendency to the results that were identified in a previous study [24]. Other studies reported that plasmonic photocatalysis of the Au-TiO_2_ combination occurred under the irradiation of visible light above 550 nm and near-infrared light according to the change in size and shape of the Au nanoparticles bonded to the TiO_2_ [40,41]. Therefore, it is reasonable that the antibacterial effect of the plasmonic photocatalyst is improved not only at 470 but also at 600 nm due to the various shapes of the fine nanoparticles [42]. 

In terms of initial cell attachment and proliferation of hMSCs cultured on various experimental specimens, we pre-tested the MTT assay. The MTT assay confirmed that the cell attachment and proliferation of hMSCs cultured on all experimental groups are similar to the values of the control group (data are not shown). 

The real-time PCR assay confirmed the highest mRNA expression values of OPN and OCN of PtAu-TiO_2_ nanotubes with the 600 nm visible light irradiation. Therefore, the osteogenic functionality of hMSCs was strongly related to (1) the wavelength of visible light irradiation and (2) the stack order of the Au and Pt coating. To better understand the correlation between the wavelength of visible light and osteogenic differentiation, we investigated the morphologies of the hMSCs cultured on the surface of the experimental specimens by FE-SEM. When the hMSCs were irradiated with 600 nm visible light, the spread of hMSCs and the number of filopodia were increased (Figure 6a). The poor attachment of the hMSCs to the surface of the specimen under 470 visible light irradiation was also strongly related to the cell proliferation and differentiation (Figure 6b). The low-level laser therapy (LLLT) effect in the region of 600–1000 nm, which plays a critical role in wound healing, tissue repair, and inflammation relief [43,44,45,46], is expected to have had a significant effect on the promoted osteogenic functionality in hMSCs. Therefore, 600 nm visible light irradiation is expected to induce the synergic effect of antibacterial activity and the osteogenic differentiation of hMSCs.

Contrary to our previous study [24], in terms of the correlation between the stack order of Au and Pt and osteogenic functionality, the experimental group in which Au was coated on the uppermost layer showed excellent osteogenic differentiation in this study. In general, Pt has been proven to prevent the electron recombination of the Au-TiO_2_ surface which promotes the plasmonic photocatalytic effect [25,26], but there are no studies on the effects of osteogenic differentiation performance in hMSCs. Several previous studies have found that the size and shape changes of Au nanoparticles strongly affect osseointegration [47,48,49]. Thus, it can be conjectured that shorter sputtering times resulted in a coating of finer nanoparticles. Finer Au and Pt nanoparticles stacked on TiO_2_ nanotubes are expected to conduct different levels of osteogenic acceleration when compared to our previous research. Further investigations will be required to investigate this phenomenon.

The alizarin red assay was performed to compare the osteogenic performance of this experimental condition (multi-coating) and the previous experimental condition (single coating). The PtAu-TiO_2_ nanotubes under visible light irradiation showed higher alizarin red values when compared to the Pt-TiO_2_ nanotubes, which showed the highest osteogenic performance in a previous study (*p* < 0.05). Therefore, the multi-coating is expected to show better osteogenic performance than a single coating.

## 5. Conclusions

Within the limitation of this study, we have confirmed through this investigation that Au and Pt multi-coatings on TiO_2_ nanotubes have excellent antibacterial activities when exposed to 470 and 600 nm visible light irradiation. This was found to be a result of plasmonic photocatalysis due to the LSPR effect of the Au and Pt nanoparticles. Moreover, the alizarin red assay showed that the multi-coating of Au and Pt is expected to improve osteogenic differentiation of hMSCs compared to the single coating of Pt. Therefore, we concluded that the TiO_2_ nanotubes multi-coated with Au and Pt can extend the limited UV antibacterial effect of TiO_2_ nanotubes into the visible light region (up to 600 nm) to improve the osteogenic performance. In addition, this novel multi-combination of noble metal nanoparticles is expected to become a base technology for the development of new surface treatment technologies for implantable devices.

## Data Availability

Not applicable.
